# Mapping the 3D remodeling of the extracellular matrix in human hypertrophic scar by multi-parametric multiphoton imaging using endogenous contrast

**DOI:** 10.1016/j.heliyon.2023.e13653

**Published:** 2023-02-13

**Authors:** Shenyi Jiang, Shuhao Qian, Lingxi Zhou, Jia Meng, Rushan Jiang, Chuncheng Wang, Xinguo Fang, Chen Yang, Zhihua Ding, Shuangmu Zhuo, Zhiyi Liu

**Affiliations:** a State Key Laboratory of Modern Optical Instrumentation, College of Optical Science and Engineering, International Research Center for Advanced Photonics, Zhejiang University, Hangzhou, Zhejiang, 310027, China; b School of Science, Jimei University, Xiamen, Fujian, 361021, China; c Jiaxing Key Laboratory of Photonic Sensing & Intelligent Imaging, Jiaxing, 314000, China; d Intelligent Optics & Photonics Research Center, Jiaxing Research Institute, Zhejiang University, Jiaxing, 314000, China

**Keywords:** Hypertrophic scar, Second harmonic generation, Two-photon excited fluorescence, Collagen, Elastin, Three-dimensional organization, Multi-parametric analysis

## Abstract

The hypertrophic scar is an aberrant form of wound healing process, whose clinical efficacy is limited by a lack of understanding of its pathophysiology. Remodeling of collagen and elastin fibers in the extracellular matrix (ECM) is closely associated with scar progression. Herein, we perform label-free multiphoton microscopy (MPM) of both fiber components from human skin specimens and propose a multi-fiber metrics (MFM) analysis model for mapping the structural remodeling of the ECM in hypertrophic scars in a highly-sensitive, three-dimensional (3D) manner. We find that both fiber components become wavier and more disorganized in scar tissues, while content accumulation is observed from elastin fibers only. The 3D MFM analysis can effectively distinguish normal and scar tissues with better than 95% in accuracy and 0.999 in the area under the curve value of the receiver operating characteristic curve. Further, unique organizational features with orderly alignment of both fibers are observed in scar-normal adjacent regions, and an optimized combination of features from 3D MFM analysis enables successful identification of all the boundaries. This imaging and analysis system uncovers the 3D architecture of the ECM in hypertrophic scars and exhibits great translational potential for evaluating scars *in vivo* and identifying individualized treatment targets.

## 1 Introduction

Hypertrophic scars are the major negative outcome in response to trauma, inflammation, surgery, or burns, and occasionally appear to occur spontaneously [1,2]. These scars are painful, hard, itchy, elevated and contracted, frequently cause significant cosmetic and symptomatic problems, and result in the loss of local function and even disability in some cases [3,4]. Clinically, hypertrophic scars can be treated in a variety of ways, including intralesional corticosteroids, topical applications, cryotherapy, surgery, laser therapy, and silicone sheeting [[5], [6], [7], [8], [9], [10], [11], [12]]. However, the outcomes of these clinical treatments are not satisfactory, largely due to a lack of understanding of the pathophysiology regarding the human hypertrophic scar [13,14]. Notably, so far few useful animal models have been developed to produce scars analogous to human hypertrophic scars. Besides, the exact boundary between hypertrophic scars and the normal skin is ambiguous, making it difficult for clinicians to remove the scar completely in plastic surgery with minimum wound to the normal skin [15]. Therefore, comprehensive information about the physiological and morphological states of skin tissues and accurate boundary identification of hypertrophic scars would enable a better understanding of the pathophysiology of this disfiguring disease and promote the therapeutic effect.

Multiphoton microscopy (MPM), such as two-photon excited fluorescence (TPEF) and second harmonic generation (SHG), shows great potential in capturing microstructures of tissue explants and living animals [16,17]. Due to the nonlinear optical effects occurring at the focal plane only, MPM has a number of unique advantages such as inherent optical slicing capability, superior tissue penetration depth, and reduced photobleaching and photodamage [18,19]. Moreover, MPM can provide label-free images of collagen and elastin fibers respectively since the non-centrosymmetric structure of collagen fibers is highly effective in generating SHG signals and elastin fibers serve as endogenous contrast for TPEF signals [20]. Benefiting from these advantages, MPM has been applied to evaluation of skin aging and photoaging, and the diagnosis of basal cell carcinoma [[21], [22], [23]]. Besides, it has become a powerful imaging tool for studying wound healing and distinguishing scars from normal skin [[24], [25], [26], [27], [28]].

Resolving structural and morphological features of collagen and elastin fibers with a high accuracy is the key to understanding their dynamic remodeling and the pathophysiology of hypertrophic scars [29,30]. However, even as high-resolution three-dimensional (3D) images of fibrous tissues became more readily accessible, quantitative analysis for their organization and morphology still largely remained limited to analysis of two-dimensional (2D) images [31,32], with only a few notable exceptions [[33], [34], [35], [36]]. The method based on polarized microscopy determined the orientation relying on the birefringence of collagen fibers, by rotating the sample slide in the azimuth and elevation planes in turn to find the two Euler angles to depict an orientation in 3D space [36]. Polarized light microscopy has been successfully applied to characterizations of fibrous structures in cartilage, brain, cervix and other tissues with a high time efficiency [[37], [38], [39]]. However, in some cases it might require picrosirius staining for enhancement of birefringence, and its determination accuracy might be affected when dealing with wavy fibers. The weighted vector summation algorithm proposed in our previous study enabled highly-accurate, truly 3D characterization of spatial orientation for fiber-like structures with a high sensitivity [40]. Based on orientation information, we further proposed optical metrics, named directional variance and waviness, respectively, as quantitative measures of alignment and bending degree of fibers, and applied them to mapping physiological and pathological functions of collagen fibers in articular cartilage, mammary glands, breast tissues, ovarian tissues and pancreas, in the context of diseases including osteoarthritis and cancer [[41], [42], [43], [44]]. Besides, we developed an algorithm for the quantification of morphologically localized distribution of fiber-like structures, termed local coverage, which provided new insights into the spatial organization of fibrous tissues [45]. Importantly, by extracting 3D information, these optical metrics faithfully reflected the real architecture of biological tissues. Further, a combination of these features might offer abundant information from complementary aspects and accelerate clinical translation of the extracellular matrix (ECM) signatures.

In this study, we collected label-free SHG images of collagen fibers and TPEF images of elastin fibers from human skin tissues, and developed a multi-fiber metrics (MFM) analysis model to uncover specific features of collagen and elastin fibers in human hypertrophic scars by integrating fiber characteristics from complementary aspects, including spatial alignment, bending degree and local fiber distribution, in a completely 3D context. Furthermore, the most discriminative features in distinguishing hypertrophic scars from normal controls were recognized and applied to the identification of the scar boundary.

## 2 Results

### 2.1 Overview of the MPM imaging and MFM analysis system

**Fig. 1:**
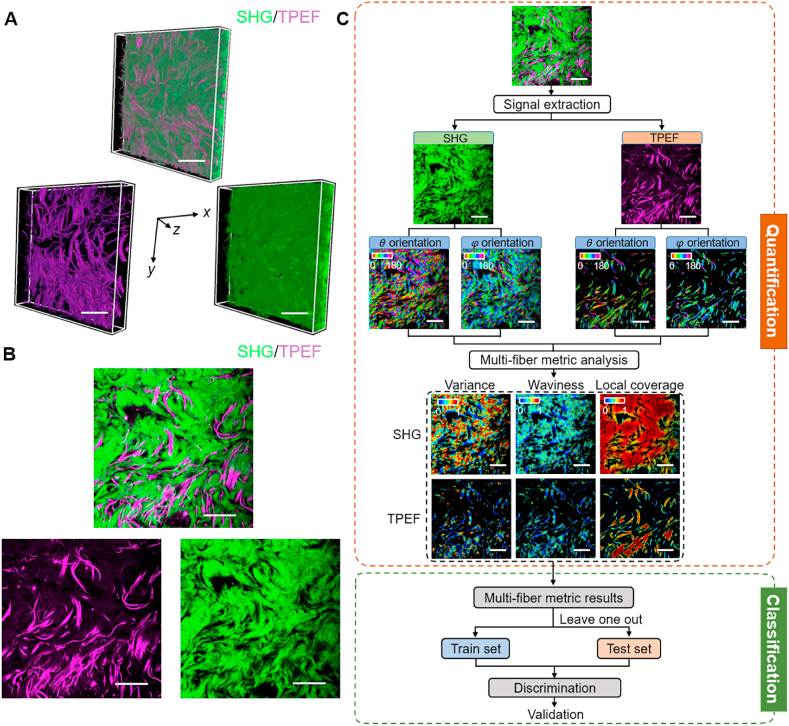
Representative MPM images of collagen and elastin fibers and flowchart for 3D MFM analysis (A and B) Representative 3D stacks (A) and corresponding 2D optical sections (B) of merged TPEF and SHG signals (top), and respective TPEF (elastin fibers, bottom left) and SHG (collagen fibers, bottom right) signals. The x, y and z axes are indicated in panel A. Scale bar: 25 μm. (C) Flowchart of the MFM analysis method and multi-parameter binary classification model. Scale bar: 25 μm. See also Figure S1.

To analyze the dynamic fiber remodeling and pathological progression of hypertrophic scars, we performed MPM on human skin specimens of hypertrophic scars and normal controls. Images of collagen fibers (Fig. 1A and B, green) were obtained by SHG imaging, and that of elastin fibers (Fig. 1A and B, magenta) were captured by TPEF imaging, without any exogenous agents. Generally, collagen fibers exhibited a dense distribution, while elastin fibers distributed sparsely and formed separate fiber bundles. These results demonstrated that MPM had great ability to reveal the fine structures of the ECM in a high-resolution and label-free manner.

These acquired MPM images were then used for the subsequent characterization of structural and morphological features for normal and hypertrophic scar tissues (Fig. 1C, top). After extracting the voxel-wise 3D orientation, as represented by the azimuthal angle θ and polar angle φ (Figure S1), directional variance and waviness, which corresponded to the alignment and the crimping level of fibrous components and ranged from 0 to 1, were calculated in a truly 3D context. A lower directional variance value corresponded to more highly aligned fibers while a higher readout revealed more random fiber alignment. For waviness, a higher value represented curvier status. Besides, the local coverage metric, ranging from 0 to 1 as well, described the voxel-wise localized distribution of fibers, with a higher value corresponding to spatially denser fiber distribution in a local area. These metrics not only provided information regarding the morphology and organization of these fiber components, but also potentially served as indicators for identifying hypertrophic scars or the boundary from normal controls (Fig. 1C, bottom).

### 2.2 3D analysis exhibits superb sensitivity in uncovering fibrous characteristics in contrast to 2D analysis

**Fig. 2:**
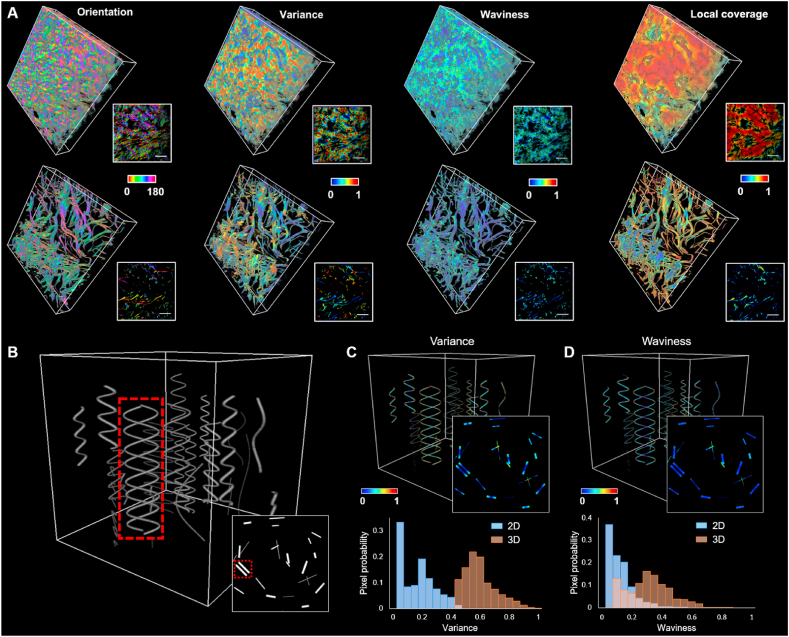
Schematic of 3D MFM analysis for collagen and elastin fibers (A) Representative 3D stacks with 2D sections of color-coded maps of orientation, directional variance, waviness and local coverage for collagen (up) and elastin (down) respectively. Scale bar: 25 μm. (B) Simulated 3D fiber stack with a number of wavy fibers, as well as the 2D projection of this stack (right bottom). ROI is marked by the red dashed box. (C) Variance maps for the 3D stack and 2D projection, along with variance distributions of the marked ROI using 2D and 3D analysis. (D) Waviness maps for the 3D stack and 2D projection, along with waviness distributions of the marked ROI using 2D and 3D analysis. See also Figure S2.

To quantitatively characterize structural and morphological features of collagen and elastin fibers, we developed a 3D MFM analysis model based on our proposed quantitative characterization algorithms. Representative 3D maps along with one certain frame from the 3D stack of these features from collagen (top) and elastin fibers (bottom) are shown in Fig. 2A. Benefiting from the voxel-wise information, probability distributions of these metrics could be extracted accordingly (Figure S2), which offered quantitative evaluation of these typical fibrous features.

In order to demonstrate the sensitivity enhancement achieved by employing a 3D analysis approach for the acquired 3D stacks, we investigated differences in resolving fibrous structures through 2D and 3D analysis respectively. To this end, we simulated a 3D stack with a number of crimping fibers (Fig. 2B), with its 2D projection shown in the inset. The comparison was carried out between the truly 3D analysis of the stack and 2D analysis of the projection at the marked region of interest (ROI, marked by dashed red box in Fig. 2B). As can be seen from variance analysis results, the alignment of these two sinusoidally shaped fibers with a phase difference of π in ROI could only be correctly resolved by 3D analysis which offered a high level of variance (i.e., low level of alignment), while they appeared as nearly parallel lines in 2D projection which led to a peak of the variance distribution at very low variance values (Fig. 2C). Similarly, the crimping feature of these fibers could only be reflected from the 3D analysis (Fig. 2D). These analysis results demonstrated that in certain cases only a truly 3D analysis could extract accurate features of fibrous structures, which might potentially lead to enhanced sensitivity in characterizing the ECM in hypertrophic scars.

### 2.3 Collagen fibers become less aligned, wavier and more sparsely distributed in hypertrophic scars

Collagen and elastin fibers were the main components of the ECM in skin tissues, which were closely related to the formation of scar, making structural and morphological features of both fiber types potential biomarkers for the hypertrophic scar progression. Here, the 3D MFM analysis model was employed to obtain the features mentioned above in hypertrophic scars, including orientation, variance, waviness and local coverage, which were compared with that from normal controls.

**Fig. 3:**
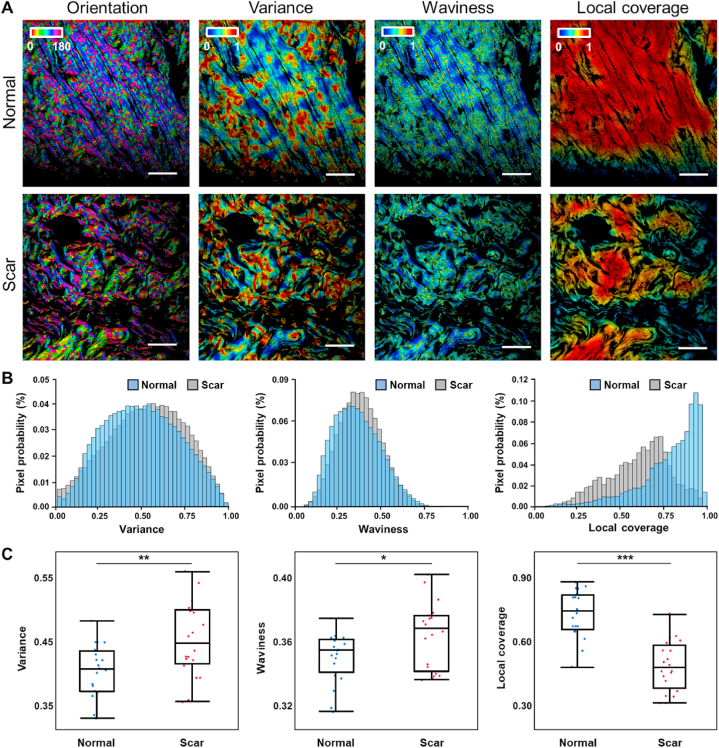
Features of collagen fibers acquired from SHG imaging and 3D MFM analysis (A) Color-coded maps of orientation, variance, waviness and local coverage in normal and hypertrophic scar tissues. Scale bar: 25 μm. (B) Probability distributions of variance, waviness and local coverage for normal (blue) and hypertrophic scar (gray) tissues. (C) Boxplots of each optical metric from normal and hypertrophic scar tissues. n = 20 specimens for both normal and scar tissues. ***, p < 0.001; **, p < 0.01; *, p < 0.05. See also Figure S3.

SHG images of collagen fibers were analyzed firstly. In terms of collagen fibers in normal skin tissues, the color-coded θ orientation map (φ map not shown due to less intuitive differences) was dominated by blue hues (Fig. 3A) corresponding to a peak at ∼130°. In contrast, orientation distribution was more random in scar tissues (Fig. 3A). For variance and waviness of collagen fibers, the readouts in normal tissue were lower than those in scar tissue, indicating that collagen fibers were relatively less aligned and wavier in hypertrophic scars (Fig. 3A and B and S3). In addition, an extreme dense fiber distribution in the normal tissue was revealed by local coverage map with dominant red hues in the map (Fig. 3A and S3) and a peak at ∼1 in probability distribution histograms of local coverage (Fig. 3B), which was in stark contrast to the lower coverage level of collagen fibers in scar tissues (Fig. 3A and B and S3). These observations from the metric maps and corresponding probability distribution histograms were consistent with statistical analysis results (Fig. 3C), quantified from 40 human skin specimens including 20 normal and 20 scar ones, and significant differences were acquired for all the three optical metrics.

### 2.4 Elastin fibers in scars show similar variations in alignment and crimping, while opposite trend in local distributions to collagen fibers

**Fig. 4:**
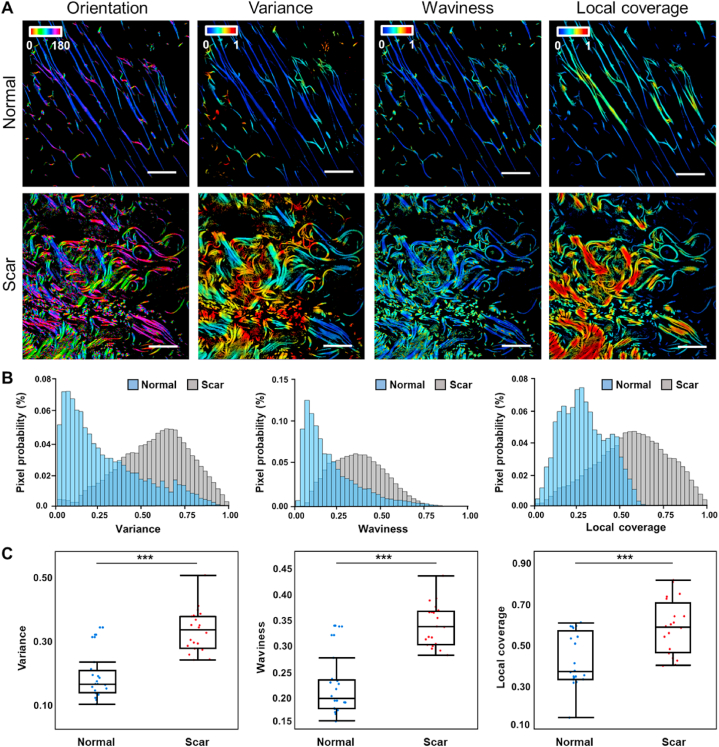
Features of elastin fibers acquired from TPEF imaging and 3D MFM analysis (A) Color-coded maps of orientation, variance, waviness and local coverage in normal and hypertrophic scar tissues. Scale bar: 25 μm. (B) Probability distributions of variance, waviness and local coverage for normal (blue) and hypertrophic scar (gray) tissues. (C) Boxplots of each optical metric from normal and hypertrophic scar tissues. *n* = 20 specimens for both normal and scar tissues. ***, p < 0.001. See also Figure S4.

The analysis for images of elastin fibers acquired from TPEF imaging was then followed. Color-coded maps from multiple parameters of elastin fibers exhibited different characteristics between hypertrophic scar and normal tissues (Fig. 4A and S4). Orientations of elastin fibers in normal tissues were extremely consistent (Fig. 4A and S4). Contrastingly, elastin orientations were disordered in hypertrophic scar tissues, as revealed by the color-coded map with a variety of hues (Fig. 4A). Similar as the readouts from collagen fibers, the variance and waviness of elastin fibers in scars were higher than those in normal controls, as evident from peaks at higher levels in the probability distribution histograms (Fig. 4B) and statistical analysis results (Fig. 4C), reflecting the degradation in alignment and increase in the crimping level of elastin fibers during the hypertrophic scar progression.

It is interesting to note that the relative variation of local coverage between hypertrophic scars and normal controls was not consistent for elastin and collagen fibers. Specifically, we observed locally denser elastin fibers from hypertrophic scar tissues (Fig. 4A, B and 4C), indicating the elastin accumulation in scars. These results demonstrated that our MFM analysis model was able to map the structural and morphological remodeling of both fiber components in a quantitative and highly-sensitive manner, and features from elastin and collagen fibers could provide complementary insights regarding the scar progression.

### 2.5 3D MFM analysis distinguishes hypertrophic scars from normal controls

Owing to the complementary nature of collagen and elastin signatures, we hypothesized that a combination of structural and morphological features from both fiber components in a certain way might offer even a more comprehensive understanding of the scar progression. To assess the independence of these optical metrics for multi-parametric combination, multicollinearity diagnostics was performed for all the data, and supported the lack of multicollinearity (Pearson product-moment correlation coefficients, |r|<0.7, Figure S5), revealing that there were no offending metrics that needed to be eliminated and each metric was independent of each other.

**Fig. 5:**
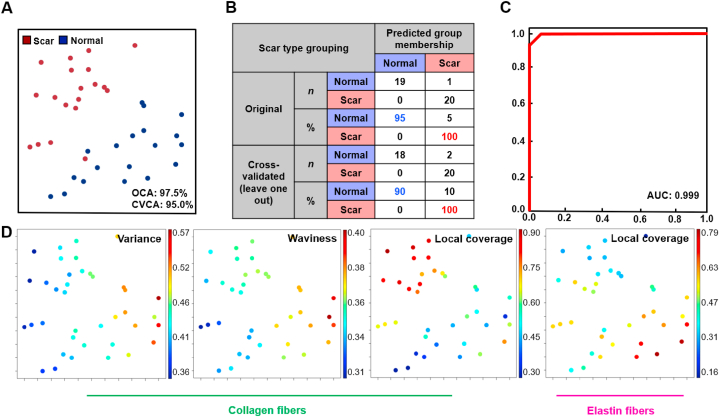
Quantitative analysis and classification of normal and hypertrophic scar tissues based on selected metrics from both collagen and elastin fibers (A) *viSNE*-based visualization of scar and normal samples based on the four selected metrics. (B) Detailed classification results of normal and hypertrophic scar tissues. (C) ROC curve of the logistic regression classifier, with the AUC value marked. (D) *viSNE* maps to show the gradient of each metric across scar and normal tissues.

To test the hypothesis, we constructed a classification model with different combinations of these six optical metrics and evaluated their discriminant ability. The performance of our classification model was estimated by the original classification accuracy (OCA) and cross-validated classification accuracy (CVCA) values. In addition, a logistic regression classifier was applied to the automatic classification, and the accuracy, sensitivity and specificity of the classifier were revealed by the area under the curve (AUC) value obtained from the receiver operating characteristic (ROC) curve. Based on the assessment of all the possible combinations (Table 1), a strategy combining directional variance, waviness and local coverage from collagen fibers and local coverage from elastin fibers led to the best classification result with OCA of 97.5%, CVCA of 95% and AUC of 0.999. It is worth mentioning that the MFM classification model integrating both collagen and elastin features performed better than any collagen-only (Figure S6) or elastin-only model (Figure S7), demonstrating the necessity of integrating features from both fiber components. Based on this MFM model, we generated scatter plot to visualize the classification of normal and scar tissues using *viSNE* analysis (Fig. 5A), which was a statistical dimension-reduction technique that enabled visualization of the clustering of features attributed to a given group by considering contributions from a number of metrics [46,47]. Detailed classification results (Fig. 5B) and corresponding ROC curve (Fig. 5C) further highlighted the strong discriminative power of the selected metric combination. In addition, we obtained *viSNE* gradient maps of different metrics to show numerical variations across the normal and scar samples (Fig. 5D). Scatters with similar hues were grouped, indicating that these selected metrics were sensitive to the identification of scars.

**Table 1:** Classification accuracy and AUC value in distinguishing normal and hypertrophic scar tissues using different combinations of fiber metric.

1	2	3	4	5	6			1	2	3	4	5	6		
●	●	●	●			OCA	92.5%	●	●			●	●	OCA	87.5%
						CVCA	92.5%							CVCA	87.5%
						AUC	0.991							AUC	0.984
●	●	●		●		OCA	95.0%		●	●	●		●	OCA	95.0%
						CVCA	92.5%							CVCA	95.0%
						AUC	0.999							AUC	0.999
●	●	●			●	OCA	**97.5%**		●	●	●	●		OCA	95.0%
						CVCA	**95.0%**							CVCA	92.5%
						AUC	**0.999**							AUC	0.999
●		●	●	●		OCA	92.5%		●	●		●	●	OCA	95.0%
						CVCA	92.5%							CVCA	95.0%
						AUC	0.991							AUC	0.999
●		●	●		●	OCA	97.5%		●	●	●	●	●	OCA	95.0%
						CVCA	95.0%							CVCA	92.5%
						AUC	0.971							AUC	0.999
●		●		●	●	OCA	97.5%	●		●	●	●	●	OCA	95.0%
						CVCA	92.5%							CVCA	92.5%
						AUC	0.991							AUC	0.991
●			●	●	●	OCA	92.5%	●	●		●	●	●	OCA	92.5%
						CVCA	92.5%							CVCA	87.5%
						AUC	0.964							AUC	0.984
		●	●	●	●	OCA	92.5%	●	●	●		●	●	OCA	95.0%
						CVCA	90.0%							CVCA	95.0%
						AUC	0.984							AUC	0.999
	●		●	●	●	OCA	92.5%	●	●	●	●		●	OCA	95.0%
						CVCA	87.5%							CVCA	95.0%
						AUC	0.974							AUC	0.999
●	●		●		●	OCA	87.5%	●	●	●	●	●		OCA	95.0%
						CVCA	87.5%							CVCA	92.5%
						AUC	0.976							AUC	0.999
●	●		●	●		OCA	92.5%	●	●	●	●	●	●	OCA	95.0%
						CVCA	92.5%							CVCA	92.5%
						AUC	0.984							AUC	0.999

*Each number corresponds to a specific feature, detailed as follows. 1: Directional variance of collagen fibers; 2: Waviness of collagen fibers; 3: Local coverage of collagen fibers; 4: Directional variance of elastin fibers; 5: Waviness of elastin fibers; 6: Local coverage of elastin fibers. See also Figures S5, S6 and S7.

### 2.6 Selective optical features successfully identify the scar boundary

**Fig. 6:**
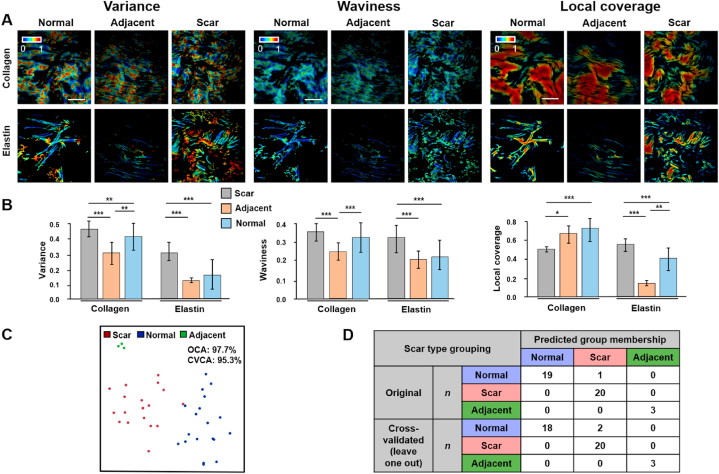
Determination of the boundary between scar and normal skin tissues using 3D MFM model (A) Representative maps of optical metrics for collagen and elastin fibers in normal, hypertrophic scar and adjacent tissues. Scale bar: 25 μm. (B) Comparison of the quantification results for each metric. Data are represented as mean ± SD.***, p < 0.001; **, p < 0.01; *, p < 0.05. (C) *viSNE* map identifying normal, hypertrophic scar and adjacent tissues generated based on 3D MFM analysis. (D) Detailed classification results of normal, hypertrophic scar and adjacent tissues.

Determination of the boundary between scar and normal skin was important for clinicians to decide the extent of the excision in surgery. The high sensitivity of our MFM analysis model in assessing the microstructure of collagen and elastin fibers provided the capability to uncover the subtle organizational variations at the border area. Moreover, the high accuracy of the selected metric combination in the classification of scar and normal samples offered further optimism for the identification of the adjacent regions. To verify this possibility, we employed our MFM analysis to quantitatively evaluate characteristics of the adjacent regions and successfully identified them from normal and scar samples using the optimum metric combination (Fig. 6). Representative maps of fiber metrics are shown in Fig. 6A, exhibiting distinct organizational and morphological features of adjacent regions compared with normal and scar ones. Different from intuition, it is interesting to note that the morphological and organizational features for both fiber components at adjacent regions were not always in some in-between status. Specifically, waviness and variance of both collagen and elastin fibers were almost the lowest in adjacent regions (Fig. 6B), revealing that fibers were especially aligned and straight at the boundary. The local coverage of elastin fibers in adjacent tissues was significantly lower than that of normal and scar tissues (Fig. 6B), while such results were not reflected in collagen fibers with a higher level of local coverage at adjacent regions than that of scar tissues, revealing the heterogeneity in organizational remodeling of these two fiber components in the ECM.

Furthermore, we carried out classification of normal, scar and adjacent sample groups using the verified metric combination that worked best for the identification of hypertrophic scar tissues from normal controls (shown in Fig. 5). As a result, an extremely high accuracy with an OCA of 97.7% and a CVCA of 95.3% was obtained (Fig. 6C), and all the adjacent samples were correctly identified (Fig. 6D), which highlighted the translational potential of our MFM model for scar boundary determination and auxiliary scar excision in surgery.

## 3 Discussion

Hypertrophic scar formation is a major clinical problem for both developing and industrialized worlds, which causes disfigurement and functional loss to the patients and leads to great physical and mental pain for them [48,49]. Clinical experience shows that hypertrophic scarring is an aberrant form of wound healing processes, accompanied by deregulation of the ECM and hypercellularity of fibroblasts which caused overactive fibrosis [[50], [51], [52]]. Collagen and elastin fibers are rich in dermis to maintain the elasticity and ductility of skin tissues, whose morphology alterations are important characteristics of hypertrophic scar progression [[53], [54], [55]]. In this work, we image collagen and elastin fibers within normal and hypertrophic scar tissues with MPM and quantitatively characterize the organizational and morphological features of both fiber components with our developed 3D MFM analysis model. As a result, the remodeling of the ECM in response to the hypertrophic scar formation is uncovered in a highly-sensitive manner, revealing the differences in 3D architecture of the ECM between normal and scar tissues, as well as heterogeneity in remodeling of these two fiber components. Collagen fibers show a well-defined orientation, high level of alignment and local coverage, and low level of crimping in normal skin tissues, consistent with previous observations from multiphoton microscopy [20,56] and transmission electron microscopy [57] on normal dermis without a scar lesion. In contrast, collagen fibers in hypertrophic scar tissues are disorganized, even disrupted, and an obvious decrease in local coverage is observed. These results are consistent with previous assessments based on immunohistochemical staining [54] and fluorescent dye (eosin) labeling [58]. Further, application of the MFM analysis model into the H&E images shows the same characteristics of local coverage reduction of collagen fibers in hypertrophic scar (Figure S8), which demonstrates the accuracy of our analysis model.

Elastin fibers are often neglected in the research of hypertrophic scar, yet in this study they are found to experience notable remodeling as well during hypertrophic scar progression and exhibit significant differences in structures between scar specimens and normal controls. Specifically, elastin fibers in normal tissues display the morphology of long ropes without obvious disruptions, acting like a spring to maintain elastic skin structures. However, in hypertrophic scar tissues, elastin fibers are typically present in fragmented status, which might explain the fact that hypertrophic scar tissues are usually hard with little elasticity [26]. These observations indicate that organizational and morphological features from elastin fibers could also potentially serve as indicators for tracking hypertrophic scar formation or for evaluating treatment responses.

The superb sensitivity of our MFM analysis model is largely attributed to the nature of truly 3D analysis. As illustrated in Fig. 2, Fig. 3D analysis exhibits significant advantages over 2D analysis by stacking up fibers in space and mimicking real tissue environments. Especially, in certain cases the fiber alignment and crimping features can only be resolved correctly by 3D analysis, which highlights the importance and necessity of this methodology in the context of fibrous structure evaluation. To the best of our knowledge, this is the first multi-parametric analysis platform for fiber-like structures that operates in a truly 3D manner. Further, the high accuracy of scar tissue classification and boundary region identification demonstrates the great translational potential of our MPM imaging and 3D MFM analysis platform.

MPM exhibits great potential in the research of hypertrophic scars especially from the capability of imaging non-fixed, unstained skin samples [59]. The signal of collagen and elastin fibers can be efficiently captured by SHG and TPEF in a label-free way and the sub-micron resolution enables high-precision evaluation of subtle organization remodeling. Moreover, depth of injury is critical to the formation of hypertrophic scar [[60], [61], [62]]; therefore, resolving patterns of wound healing and severity of the scarring and 3D imaging capacity offered by MPM enable assessments of the deep-layer remodeling of the ECM and thus a better understanding of hypertrophic scar formation and progression. It should be pointed out that the thickness of human skin, which is over 1 mm, is still a challenge for the maximum imaging depth of MPM [63]. Fortunately, the introduction of the coherent imaging method could amplify the weak signal at large depth and enhance the signal-to-noise ratio of the MPM for imaging the intact skin tissues [[64], [65], [66]], which makes it possible to realize full-depth, *in vivo*, non-invasive skin detection directly on a patient and provides further optimism for the translational application of MPM on the dermatological research.

Accurate identification of the boundary between normal skin and hypertrophic scar is challenging in surgery since clinicians often rely on their experience to decide the extent and scope of removal, possibly leading to inevitable inaccuracy and subjectivity. Both over- and under-excision of the scar would have adverse effects on the patient. Especially, under-excision would cause incomplete removal of scars which might lead to recurrence [[67], [68], [69]]. The high resolution offered by MPM could significantly improve the accuracy of boundary determination and the quantitative outputs from our developed 3D MFM model could provide objective criteria to identify and assess the boundary region. As a result, all the adjacent regions are successfully identified in this study. Moreover, the rapidity of MPM and the high time-efficiency of our MFM analysis ensure the potential of real-time feedback and assessments of scar-skin boundaries in surgery.

In conclusion, by combining high-resolution, label-free MPM imaging with MFM analysis, we propose a promising approach for uncovering the remodeling of the ECM and identification of hypertrophic scar and/or boundary using organizational and morphological features of collagen and elastin fibers as quantitative indicators. MPM is performed on human skin specimens to capture 3D microstructure of collagen and elastin fibers, and MFM analysis model is constructed to realize quantitative, truly 3D assessment of hypertrophic scar progression based on highly-sensitive features from both fiber components. Linear discrimination analysis of hypertrophic scar and normal skin tissues is carried out using outputs from MFM analysis and a final accuracy better than 95% is obtained, highlighting the sensitivity of the multi-parametric model. The successful application of MFM analysis in scar boundary identification reveals unique fiber structural features of orderly aligned collagen and elastin fibers at borders, and demonstrates the translational potential of this imaging and analysis platform. Compared with previous studies on hypertrophic scar [26,54,58], MFM analysis provides voxel-wise characterizations in an automatic way, and reveals the structural and morphological differences of collagen and elastin fibers between normal and hypertrophic scar tissues in a truly 3D manner which faithfully reflects the real architecture of biological tissues, demonstrating great translational possibility due to the high accuracy of scar tissue classification and boundary region identification. As MPM becomes more readily accessible, our methodology will offer a unique potential to assess *in vivo* scar development and the impact of new treatment targets. Especially, we will focus on the clinical translation of this methodology by developing hand-held probe based MPM system for convenient and real-time monitoring of the occurrence and development of hypertrophic scar *in situ* and *in vivo* [[70], [71], [72]]. Moreover, mitochondria play an important role in maintaining the homeostasis of biological tissues and the dysfunction of mitochondria may also be a non-negligible indicator of scarring [73,74]. With the development of MPM as a new non-invasive, high-resolution and label-free method to study mitochondrial metabolism [75], we will apply MPM to metabolic characterizations of mitochondria in skin tissues in the next move to further understand the pathophysiology of human hypertrophic scar, so as to provide insights from not only the ECM [76] but also cells [77].

## 4 Limitations of the study

As a preliminary investigation, the number of the adjacent specimens is very limited. However, our findings regarding the specific morphological structures at the boundary and the heterogeneity in organizational remodeling of the two fiber components are interesting, and the correct identification of all the adjacent specimens is highly promising. Clearly, more extensive studies with increased number of specimens are needed in our future work to validate these observations and assess the capability of our methodology in boundary identification.

## Star methods

### Resource availability

#### Lead contact

Further information and requests for resources and reagents should be directed to and will be fulfilled by the lead contact, Zhiyi Liu (liuzhiyi07@zju.edu.cn).

## Materials availability

This study did not generate new unique reagents.

## Data and code availability

The data that support the findings of this study are available from the corresponding author upon reasonable request. The code for analysis is available at: https://github.com/ShuhaoQian/Waviness_calculation_code.

## Experimental model and subject details

### Human skin specimens

Human hypertrophic scar specimens and normal skin tissues used in this study were collected from 20 patients aged from 10 to 50 years old who had undergone skin plastic surgery. The plastic surgery was done mainly 1–2 years after the formation of the scar, which allowed enough time for the scar to fully heal and possibly shrink and flatten on its own. Informed consent was obtained from every patient who participated in the study. All the procedures were executed strictly following the institutional regulations of Zhejiang University regarding clinical investigation of human subjects in biomedical research.

Once the skin samples were excised from the patients, we snap-froze and stored them immediately in liquid nitrogen (−196 °C). Prior to imaging, we cut the tissue sections into ∼30 μm thickness and sandwiched them with microscope slides and a cover glass for imaging. A small amount of phosphate-buffered saline (PBS) solution was dropped into tissue specimens to avoid shrinkage or dehydration during imaging process. In this study, we imaged and analyzed 40 tissue section samples (20 scar ones and 20 normal ones) from 20 patients. The imaged regions were marked after the imaging procedure and then prepared for the pathological staining. Tissue types (normal, scar or adjacent) of the imaged regions were confirmed by experienced pathologists according to the staining readouts. If the imaged fields were not representative of the corresponding tissue type, then these fields would not be used for the subsequent evaluation.

### Multiphoton microscopy (MPM) of tissue specimens

The MPM system used in this study was comprised of three parts: an excitation light source (Coherent Mira 900-F; Coherent Inc., Santa Clara, CA, U.S.A.), a high-throughput scanning inverted microscope (Zeiss Axiovert 200; Carl Zeiss MicroImaging, Jena, Germany) and a spectral imaging detection system (META detector; Zeiss), as described in our previous study [78]. Specifically, the excitation light source was a mode-locked Ti:sapphire laser with a pulse width of 110 fs and a repetition rate of 76 MHz. Excitation wavelength was tunable from 700 to 980 nm and the average power of laser was controlled below 6 mW. It was worth noting that the choice of laser power was a matter of careful consideration because a high power can lead to cell damage [79,80] and 15 mW was the measured safe power threshold on skin for the near-infrared laser used in our experiment. Furthermore, we increased the power and found that the skin started to burn when it reached about 60 mW in our system settings. An acoustic-optic modulator was used to adjust the intensity of the femtosecond laser beam before it entered the microscope and the polarization direction of the laser light was circular. A galvanometer driven optical scanner (Zeiss) was used to scan the beam in the focal plane. The objective applied in all experiments was an oil immersion objective with a large aperture (Plan-Apochromat 63 × , NA1.4; Zeiss). A dichroic beam splitter was utilized to reflect the incident light beam to the sample plate and transmit the TPEF and SHG signals generated by the sample to the META detector. The META detector consisted of a reflective grating and a 32-channel photomultiplier tube (PMT) array for the acquisition of TPEF and SHG signals.

In this study, 850 nm was used as the excitation wavelength for both SHG and TPEF imaging. High-resolution, high-contrast images of collagen and elastin fibers were extracted in different channels according to their emission spectrum range. Specifically, we used one channel covering the wavelength range from 414 to 436 nm to display the morphology of collagen fibers using SHG signals, and the other channel corresponding to the wavelength range of 457–714 nm to represent the microstructure of elastin fibers using TPEF signals.

The imaging depth of our system for skin tissues was tested to be ∼250 μm. Therefore, our system was able to acquire images throughout the entire tissue section with ∼30 μm in thickness. We obtained one 3D image stack (512 × 512 × 105 voxels, 206 × 206 × 31 μm) from each tissue section (*i.e.*, 20 scar ones and 20 normal ones) for the subsequent analysis. In addition, we took three image stacks from adjacent regions between scar and normal skin tissue from three independent scar specimens. As mentioned above, all the imaged fields were further validated by pathologists by checking with staining readouts.

### 3D multi-fiber metrics (MFM) analysis model

To quantitatively characterize the structural and morphological features of collagen and elastin fibers in normal and hypertrophic scar tissues, we developed 3D MFM analysis model based on our previously proposed quantification algorithms to acquire four quantitative metrics termed orientation, directional variance, waviness and local coverage for assessment of collagen and elastin fibers. Firstly, we obtained the voxel-wise 3D orientation of fibers using the weighted vector summation algorithm, as detailed in our previous study [40]. Briefly, 3D orientation was defined by the azimuthal angle θ and the polar angle φ (Figure S1). The polar angle was typically difficult to calculate. To address this issue, two extra angles, β and γ, were introduced in the way similar to the azimuthal angle θ, with β defined as the angle between the fiber's projection on the *zx* plane and the *x* axis, and γ defined as the angle between the fiber's projection on the *yz* plane and the *-y* axis (Figure S1). With the extraction of these two extra angles, the polar angle φ could be resolved by the equation:

(1) $$\tan^{2}\;\varphi =\frac{1}{\tan^{2}\;\gamma }+\frac{1}{\tan^{2}\;\beta }$$

The general process of MFM analysis is shown in Fig. 1C. Based on the voxel-wise 3D orientation, directional variance and waviness, quantified in a truly 3D context, were calculated to measure the fiber alignment and bending degree [[41], [42], [43], [44]], with both metrics ranging from 0 to 1. For directional variance, a lower variance value corresponded to more highly aligned fibers while a higher readout revealed more random fiber alignment. For waviness, a higher value represented curvier status. Local coverage was another metric that quantified the voxel-wise localized distribution of fibers [45]. The value of local coverage also ranged from 0 to 1 with a higher value corresponding to morphologically denser fiber distribution in a local area. Based on voxel-wise quantities, we were able to generate color-coded maps to visually display each metric. Algorithm details of these metrics mentioned above could be found in our previous study [[40], [41], [42], [43], [44]] and all the calculations were performed using MATLAB software.

### *viSNE* for visualization of MFM analysis results

The *viSNE* dimensionality reduction method was performed to visualize MFM results based on features of collagen and elastin fibers, including directional variance, waviness and local coverage from both components. *viSNE* method was initially developed for dealing with cellular heterogeneity issues [46,47], but we extended it to a more general application. *viSNE* utilized *t*-distributed stochastic neighbor embedding (*t-SNE*) method to minimize the differences between high-dimensional and low-dimensional space and generated a 2D scatter map of points (different imaged fields in this study) with different labels (normal, scar and adjacent). Briefly, a pairwise distance matrix was calculated in high-dimensional space and then converted into a low-dimensional similarity matrix. All the points were randomly mapped in the low-dimensional space and then iterated to minimize the difference between high- and low-dimensional similarity matrices. *viSNE* dimensionality reduction process and *viSNE* maps were performed and generated in Cytobank (www.cytobank.org).

## Statistical analysis

Statistical analysis was performed using SPSS software. Data were represented by mean and standard deviation (mean ± SD). For quantitative comparison of normal and hypertrophic scar groups, student's *t*-test was performed. A one-way ANOVA post-hoc Tukey HSD test was performed to assess differences among normal, scar and adjacent skin tissues. Differences were considered statistically significant at p < 0.05.

Linear discriminant analysis was performed based on these quantitative metrics to classify hypertrophic scar, adjacent and normal skin tissues using SPSS. To investigate the influence of different metrics on the discriminant results of the classification, original classification accuracy (OCA) and cross-validated accuracy (CVCA) were obtained respectively based on all data sets and leave-one-out cross-validation scheme. Logic regression analysis was further carried out to assess the discriminant ability provided by different combinations of metrics. By drawing the true positive rate against the false positive rate, we obtained a receiver operator characteristic (ROC) curve and calculated the area under the curve (AUC) value as an indicator to evaluate the performance of the classifier.

## Author contribution statement

Shenyi Jiang; Shuhao Qian; Lingxi Zhou; Jia Meng; Rushan Jiang; Chuncheng Wang; Xinguo Fang; Chen Yang: Performed the experiments; Analyzed and interpreted the data; Contributed reagents, materials, analysis tools or data; Wrote the paper.

Zhihua Ding; Shuangmu Zhuo; Zhiyi Liu: Conceived and designed the experiments; Analyzed and interpreted the data; Contributed reagents, materials, analysis tools or data; Wrote the paper.

## Funding statement

Zhiyi Liu was supported by 10.13039/501100012166National Key Research and Development Program of China [2019YFE0113700 & 2017YFA0700501], 10.13039/501100004731Natural Science Foundation of Zhejiang Province [LR20F050001], National Natural Science Foundation of China [62275232; 61905214; 62035011; 11974310; 31927801].

## Data availability statement

Data will be made available on request.

## Declaration of interest's statement

The authors declare no competing interests.
