# Supplementary material for: Mapping the 3D remodeling of the extracellular matrix in human hypertrophic scar by multi-parametric multiphoton imaging using endogenous contrast
Source: Heliyon. 2023 Feb 13;9(2):e13653. doi: 10.1016/j.heliyon.2023.e13653 (PMC9975259; doi:10.1016/j.heliyon.2023.e13653)
Supplement: Supplemental information [file mmc1.docx]

Supplemental information

**Mapping the 3D remodeling of the extracellular matrix in human hypertrophic scar by multi-parametric multiphoton imaging using endogenous contrast**

*Shenyi Jiang,^1, †^ Shuhao Qian,^1,†^ Lingxi Zhou,^1^ Jia Meng,^1^ Rushan Jiang,^1^ Chuncheng Wang,^1^ Xinguo Fang,^1^ Chen Yang,^1^ Zhihua Ding,^1^ Shuangmu Zhuo,^2,*^ Zhiyi Liu,^1,3,4,5,*^*

^1^State Key Laboratory of Modern Optical Instrumentation, College of Optical Science and Engineering; International Research Center for Advanced Photonics, Zhejiang University, Hangzhou, Zhejiang 310027, China

^2^School of Science, Jimei University, Xiamen, Fujian 361021, China

^3^Jiaxing Key Laboratory of Photonic Sensing & Intelligent Imaging, Jiaxing 314000, China

^4^Intelligent Optics & Photonics Research Center, Jiaxing Research Institute, Zhejiang University, Jiaxing 314000, China

^5^Lead contact

^†^These authors contributed equally to this work

*Correspondence: liuzhiyi07@zju.edu.cn (Z.L.), shuangmuzhuo@gmail.com (S.Z.)

**
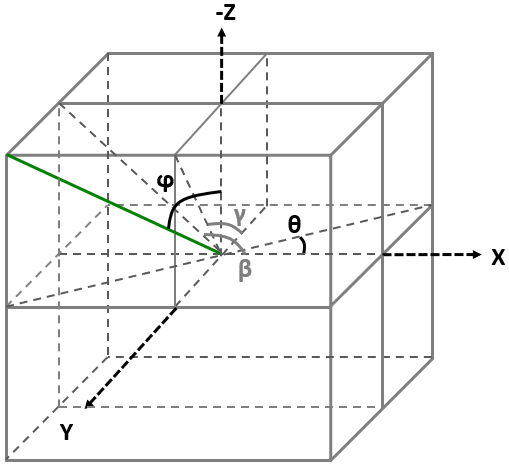
**

**Figure S1. Definition of angles to describe a certain orientation (the green line) in 3D space.** Related to Figure 1.


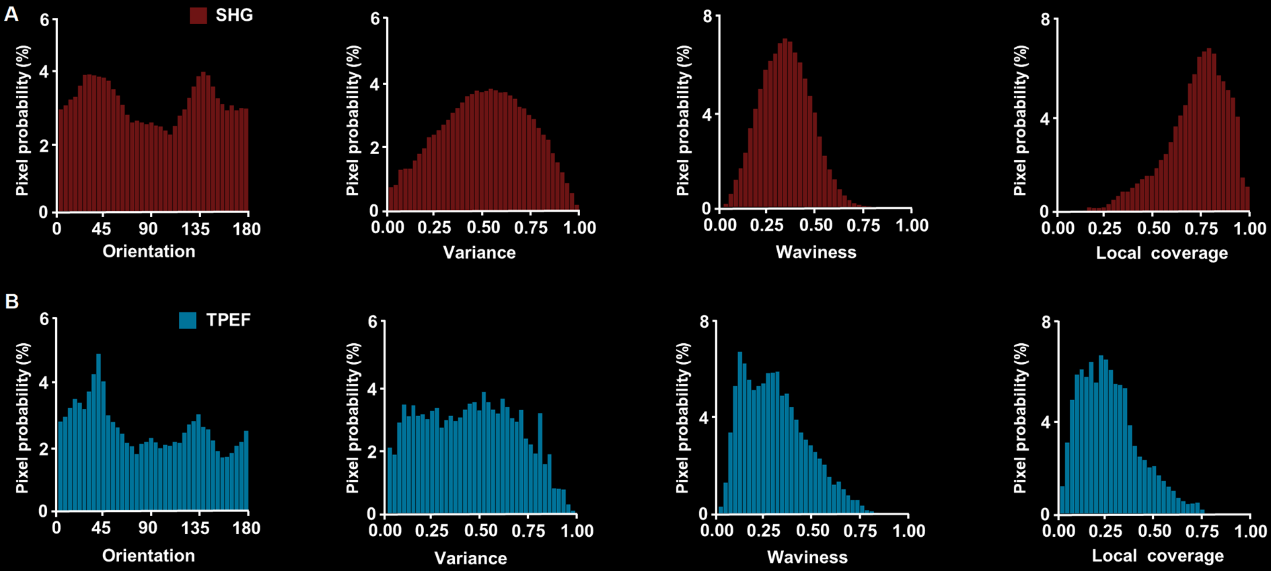


**Figure S2. Distribution histograms of different optical metrics from different fiber components.** (A) Distribution histograms of metrics from collagen fibers. (B) Distribution histograms of metrics from elastin fibers. Related to Figure 2.


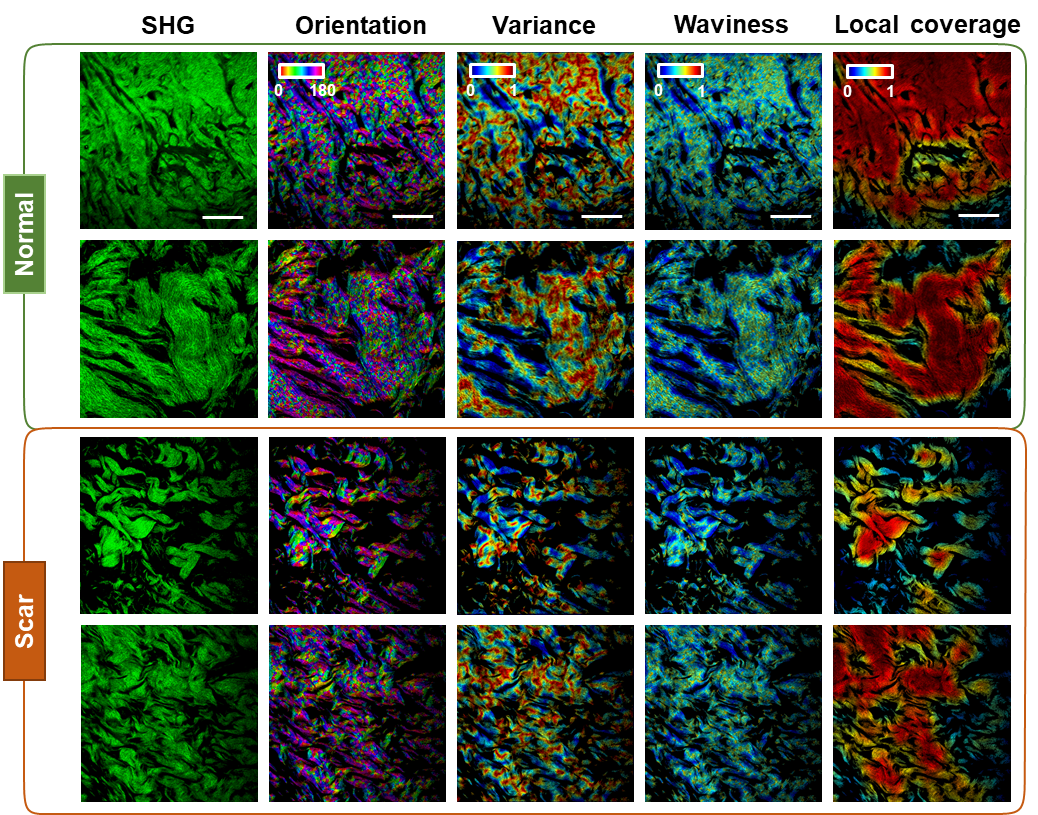


**Figure S3. More examples of morphological features of collagen fibers acquired from the 3D MFM analysis model.** Scale bar: 25 μm. Related to Figure 3.


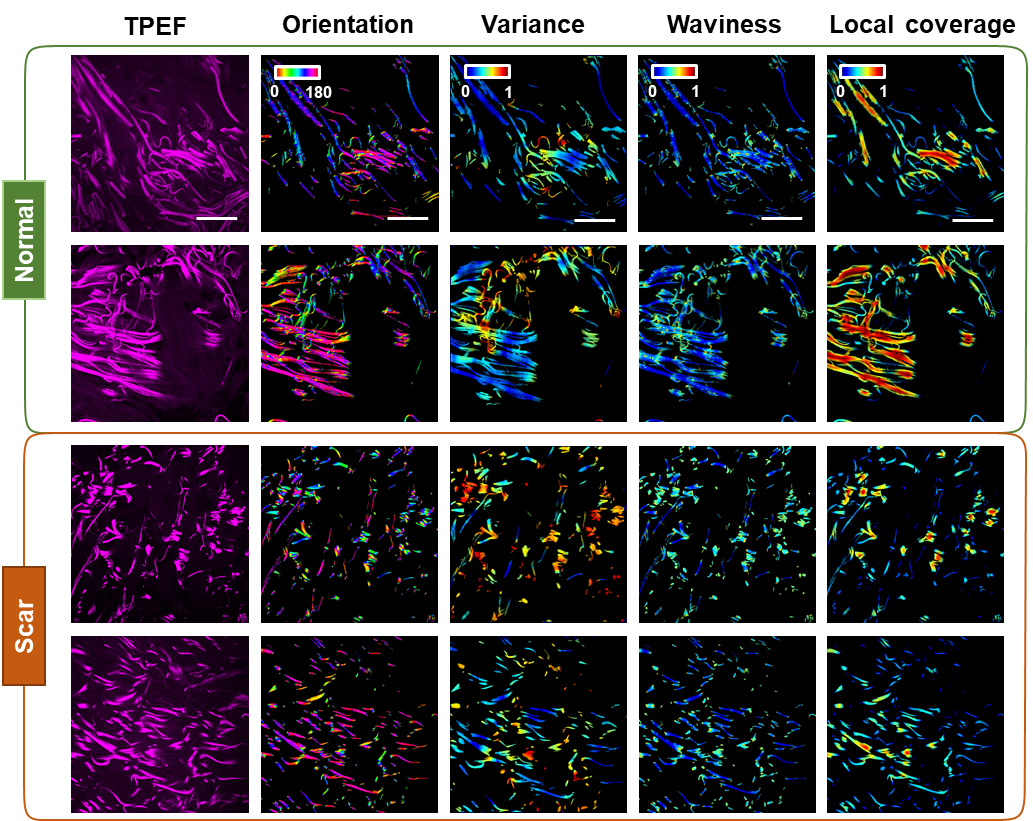


**Figure S4. More examples of morphological features of elastin fibers acquired from the 3D MFM analysis model.** Scale bar: 25 μm. Related to Figure 4.


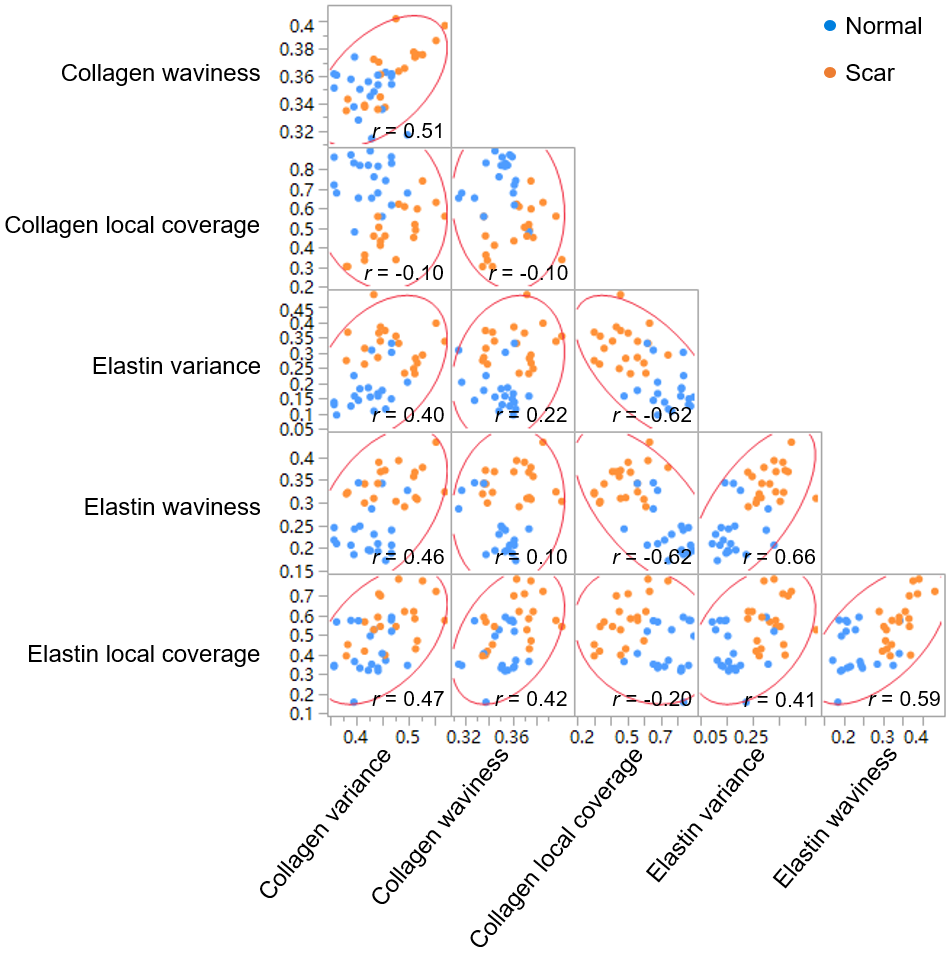


**Figure S5.** **Multicollinearity diagnostics through pairwise correlations from all the data**. All the correlation coefficients (marked in each panel) satisfy the equation: $\left|r\right|<0.7$, indicating no offending variables and independence among these six variables. Related to Table 1.

**
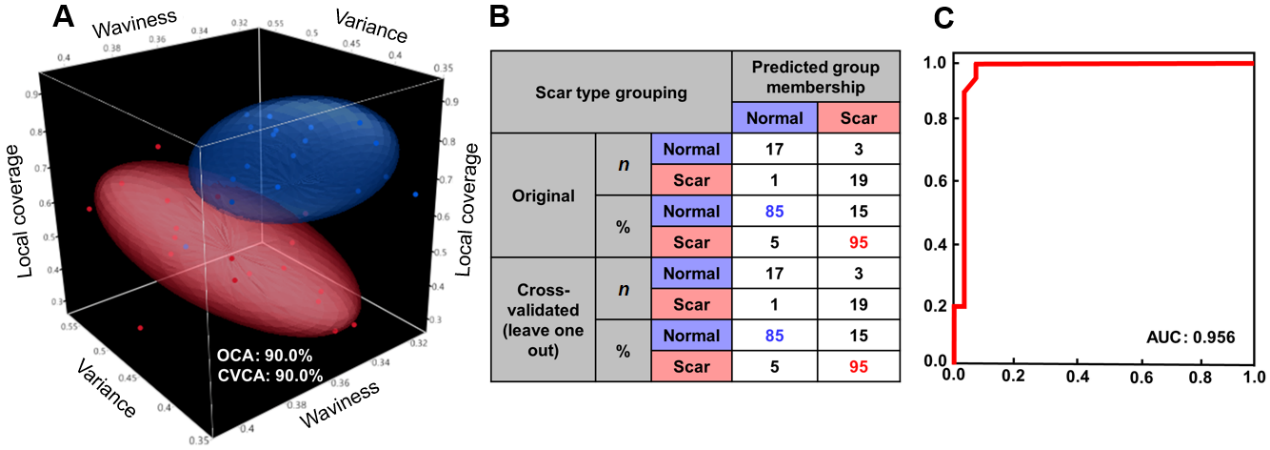
**

**Figure S6. Classification of normal and hypertrophic scar tissues based on morphological and structural features from collagen fibers.** (A) 3D scatterplot showing the classification of normal and scar tissues. (B) Classification results. (C) ROC curve of the logistic regression classifier, with the AUC value marked in the curve. Related to Table 1.


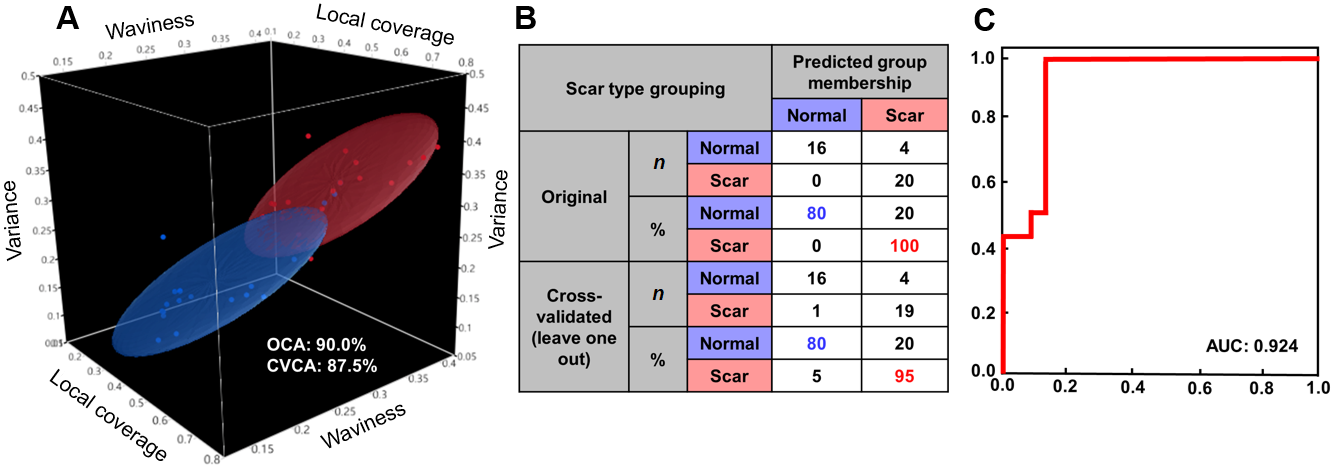


**Figure S7. Classification of normal and hypertrophic scar tissues based on morphological and structural features from elastin fibers.** (A) 3D scatterplot showing the classification of normal and scar tissues. (B) Classification results. (C) ROC curve of the logistic regression classifier, with the AUC value marked in the curve. Related to Table 1.


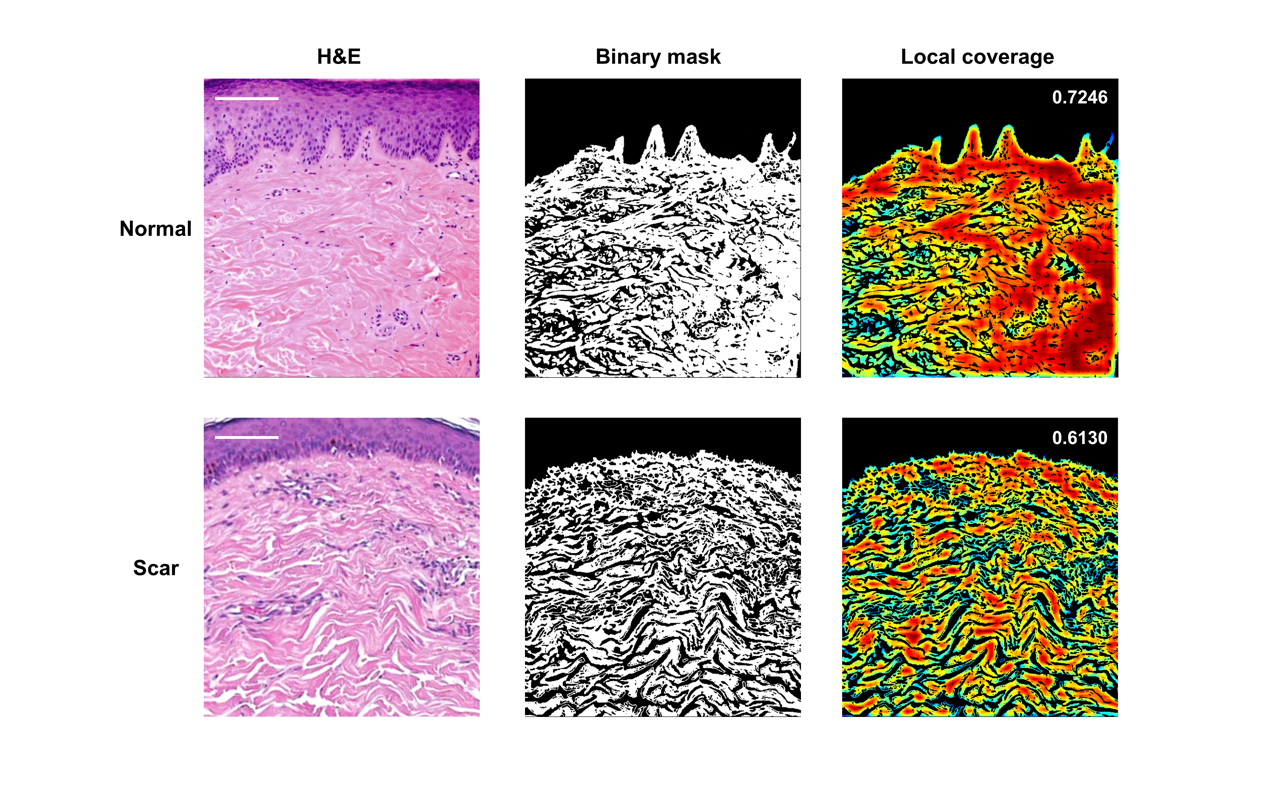


**Figure S8.** **Characterizations of local coverage of collagen fibers within representative hematoxylin-eosin (H&E) staining images of normal and hypertrophic scar tissues.** Left: H&E images. Middle: the binary mask of collagen fibers within the H&E images. Right: the corresponding local coverage maps, with the calculated local coverage values indicated in the maps. Scale bar: 100 μm.
